# Molecular Dynamics Simulation of the Interaction between Graphene Oxide Quantum Dots and DNA Fragment

**DOI:** 10.3390/ma15238506

**Published:** 2022-11-29

**Authors:** Lingxiao Wu, Pengzhen Zhang, Hanxing Zhou, Jing Li, Xin Shen, Tianyu Li, Zhe Kong, Wei Hu, Yongjun Zhang

**Affiliations:** 1 Center of Advanced Optoelectronic Materials and Devices, Key Laboratory of Novel Materials for Sensor of Zhejiang Province, College of Materials and Environmental Engineering, Hangzhou Dianzi University, Hangzhou 310018, China; 2 College of Automation, Hangzhou Dianzi University, Hangzhou 310018, China; 3 School of Medicine, Hangzhou Normal University, Hangzhou 311121, China; 4 Shandong Provincial Key Laboratory of Molecular Engineering, School of Chemistry and Pharmaceutical Engineering, Qilu University of Technology (Shandong Academy of Sciences), Shandong 250353, China

**Keywords:** molecular dynamic simulation, graphene oxide quantum dots, biosafety, DNA

## Abstract

Due to their excellent physical properties, graphene oxide quantum dots (GOQDs) are widely used in various fields, especially biomedicine. However, due to the short study period, their biosafety and potential genotoxicity to human and animal cells are not well elucidated. In this study, the adsorption of GOQDs with different concentrations and oxidation degrees on DNA was investigated using a molecular dynamics simulation method. The toxicity to DNA depended on the interaction mechanism that GOQDs adsorbed on DNA fragments, especially in the minor groove of DNA. When the number of the adsorbed GOQDs in the minor groove of DNA is small, the GOQD inserts into the interior of the base pair. When there are more GOQDs in the minor groove of DNA, the base pairs at the adsorption sites of DNA unwind directly. This interaction way damaged the double helix structure of DNA seriously. We also compare the different functional groups of -1COOH. The results show that the interaction energy between 1COOH-GQD and DNA is stronger than that between 1OH-GQD and DNA. However, the damage to DNA is the opposite. These findings deepen our understanding of graphene nanotoxicity in general.

## 1. Introduction

Graphene oxide (GO) is a special carbon material with rich oxygen-containing functional groups such as epoxides, carbonyl groups, carboxyl groups, and hydroxyl groups [1,2,3]. Compared with pristine graphene (PG), which has a strong hydrophobic effect, GO has good hydrophilicity due to a large number of carboxyl groups and hydroxyl groups on the surface, which can be evenly dispersed in aqueous solution to form a stable colloid solution [2,4,5,6]. Due to their excellent optical, mechanical, and electrical properties, GO nanomaterials are widely used in sensing, aerospace, new energy, disease diagnosis, and other aspects [7,8,9,10]. GO quantum dots (GOQDs) inherit many unique properties of GO; however, their size is less than 100 nm [11]. Their applications are not limited by their band gap [12,13]. GOQDs can be designed by abundant surface functionalities and edge effects [14,15,16]. Zhu et al. prepared an electrochemical biosensor modified with graphene oxide quantum dots/carboxylated multi-walled carbon nanotubes (GOQDs/CMWCNTs) hybrid material. The toxicity was evaluated via the changes in the electrochemical signal derived from the nucleotide catabolism of cells [17]. Wang et al. found that GOQDs can also be used as vectors to deliver protein or drug molecules to cells because of their high surface area [18]. Shi et al. reported the design and synthesis of multifunctional biocompatible anti-Glypican-3-antibody-attached GOQDs-decorated magnetic nanoplatforms that can deliver targeted capture of cancer cells from an infected blood sample, followed by accurate analysis using two-photon imaging [19]. Choi et al. demonstrated that the upconversion nanoparticles with GOQD shell loaded with hypocrellin A could be excellent candidates as multifunctional agents for cell imaging, drug delivery, and cell therapy [20]. In the experiment, Ji et al. reported GOQDs blocked autophagic flux by decreasing the amount and enzymatic activity of cathepsin B and inhibiting lysosome proteolytic capacity in GC-2 and TM4 cells, which might have a potential hazard to male reproduction [21]. Li et al. reported GOQDs induced DNA damage and disrupted microtubule structure in human esophageal epithelial cells [22]. However, the interaction mechanism between GOQDs and DNA is still unclear. Therefore, understanding the toxicity of GOQDs is conducive to promoting the application of GOQDs in biomedicine.

In order to verify whether GOQDs can affect living organisms for biomedical applications, it is necessary to consider their biosafety, cytotoxicity, and genotoxicity. GOQDs can be unintentionally inhaled or intentionally injected and implanted as a part of new biomedical technologies. Lou et al. reported that GOQDs could enter cells through various mechanisms, including endocytosis, pinocytosis, and phagocytosis [3]. In recent years, the genotoxicity of GO has been extensively evaluated through both theoretical and experimental studies [23,24,25,26]. Due to the different degrees and types of surface oxidation and chemical properties, it may show lower toxicity than PG in some experiments and higher cytotoxicity in others [22,27,28,29]. For instance, Chong et al. established that GO has less cytotoxic than pure graphene, which is consistent with the results of Liao [26,30]. Akhavan et al. demonstrated that GO nanowalls reduced by hydrazine are more toxic to bacteria than unreduced GO nanowalls [31]. In previous studies, the adsorption or self-assembly of single- or double-stranded DNA of different lengths on two-dimensional carbon materials, such as GO and carbon nanotube arrays, was studied. Zeng et al. established that GO has better adsorption capacity and indicated that GO would produce more toxic effects on DNA by comparing the binding sites of double-stranded DNA on GO with those on PG [32,33]. Fang et al. established that there are large unoxidized and oxidized regions on GO, which made hydrophilic and hydrophobic properties coexist in the boundary region. Thus, the hydrogen bonds in this region were very active and easy to break or form, providing stronger adsorption for single-stranded DNA [16]. However, during the interaction with DNA, proteins, and other biomolecules, a larger GO often leads to DNA adsorption on its surface. Related studies have also shown that small molecules interact with DNA in diverse ways, including covalent bond binding, non-covalent bond binding, and shear interaction. For instance, Liu et al. established that benzo[a]pyrene-7,8-dioneo could occupy the binding site of DNA transcription and affect the expression of its genetic information using first principle calculation [34]. Bu et al. established that ciprofloxacin could enter the small groove position of DNA in an embedded way [35]. Geng et al. reported that graphene quantum dots (GQDs) could induce DNA damage and cell apoptosis in various sensitive and resistant cancer cell lines [36]. Ou et al. demonstrated that the surface oxygen-containing groups of GO play essential roles in the reduced genotoxicity of graphene [37]. However, the interaction between GOQDs and DNA at the molecular level remains unclear. In particular, the effect of the different functional groups of GOQDs on DNA damage is unclear. This significantly limits the practical application of GOQDs in the biomedical field.

In addition to experiments, molecular dynamics (MD) simulations have been widely applied in the field of biological nanotechnology. It can provide details of the interaction between biomolecules and inorganic materials at the atomic level, play a good role in controlling the variables of inorganic materials, and can be a good supplement to experimental data [38,39,40,41,42]. Zhou et al. explored the interaction mechanism between GQDs and proteins of different sizes and analyzed the size effect of GQDs on protein adsorption [43]. In our previous work, the interaction between the size of the GQDs and DNA was investigated through MD simulations [44]. In this study, the interactions between GOQDs and DNA fragments were studied. In particular, the effects of functional groups and the oxidative degree of GOQDs on the interaction of DNA were explored.

## 2. Computational Details

### 2.1. System Setup

**Figure 1:**
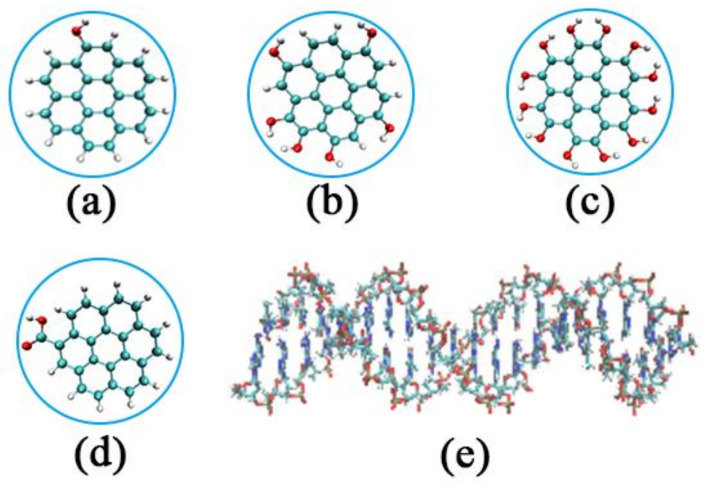
Initial structure of GOQDs and DNA fragment: (**a**) 1OH-GQD; (**b**) 6OH-GQD; (**c**) 12OH-GQD; (**d**) 1COOH-GQD; (**e**) poly(A-T)_20_.

The structures of GOQDs can be treated as benzene rings connected by carbon atoms. In our simulation, the geometry of all GOQDs was circular, and the size of the GOQDs was defined according to the number of carbon rings. The initial coordinates of the carbon atoms around the central carbon atom were x^2^ + y^2^ < R^2^, where R is the radius of the GOQDs. The unsaturated carbon at the edge of all GOQDs was connected to an H atom, and then its edge was randomly oxidized. Finally, we used Gaussian 09 D.01 software (sourced from Jinan, China) to optimize the GOQDs structure, and the optimized GOQDs structure was used as the initial structure for the MD simulation [45]. We selected GOQDs with seven carbon rings and random distributions of 1OH, 6OH, and 12OH on the edges using a home script. As shown in Figure 1a–c, the systems were named 1OH-GQD, 6OH-GQD, and 12OH-GQD, respectively.

DNA molecules with A-T bases were constructed using Hyperchem software. Subsequently, the DNA fragment molecules were balanced with *NPT* for 20 ns, and the balanced configuration was taken as the initial configuration, as shown in Figure 1e.

**Figure 2:**
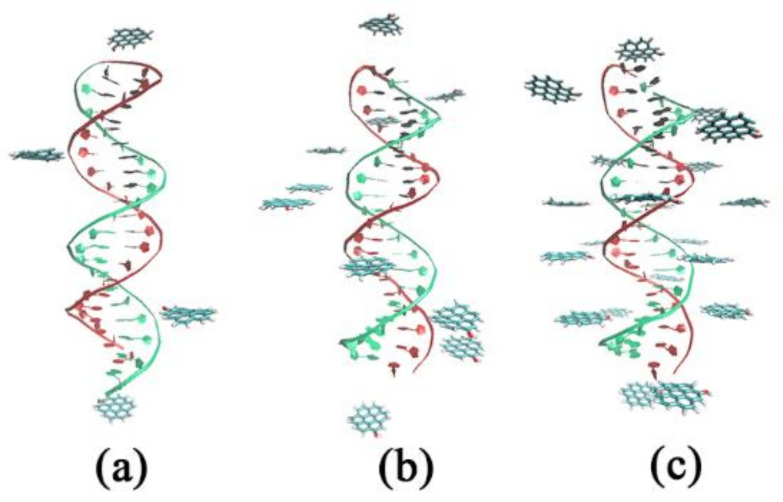
Position relationship between various GOQDs and DNA before MD: (**a**) the number of GOQDs is 4; (**b**) the number of GOQDs is 10; (**c**) the number of GOQDs is 20.

The initial system configurations are shown in Figure 2. The distance between two GOQDs in the head and the terminal is ca. 8 nm, except for the two GOQDs; the others were evenly distributed around the DNA and with a distance of approximately 0.2 nm from the DNA. All the sizes of the simulation systems were 11.0 × 11.0 × 11.0 nm^3^. Detailed information on the simulation systems is presented in Table 1.

### 2.2. MD Simulations

Gromacs 5.0.4 software package was used to complete all simulations. All the MD simulations were based on the AMBER03 force field [46,47]. The harmonic bond potentials of C–C and C–H bonds of GOQD, harmonic angles of C–C–H and C–C–C bonds, potential parameters of harmonic dihedral angles, and nonbond Lennard Jones parameters were derived from the work of Cohen–Tanugi et al. [48]. All bonds, including those bonded to H atoms, were constrained using the LINCS algorithm. The system was dissolved in TIP3P water in the simulation box. In the simulation, the Nose–Hoover thermostatic method was used to maintain the temperature at 310 K, and the pressure was maintained at 1 bar using the isotropic Parrinello–Rahman pressure regulator. All systems were simulated with the NPT ensemble for 100 ns, the cutoff for the nonbonded Van der Waals interaction was 12 Å, and the time step was 2 fs. The instantaneous structure diagram of the simulation process was visualized using the VMD software.

## 3. Results and Discussion

### 3.1. Effects of Different Oxidation Degrees of GOQDs on the Adsorption Sites of DNA

**Figure 3:**
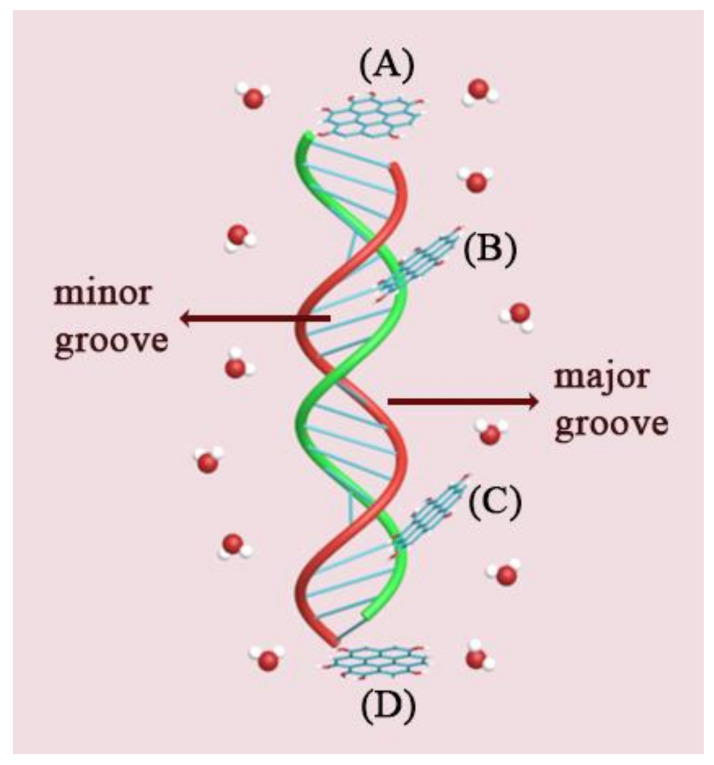
Snapshots of absorption of various GOQDs on DNA fragment in four sites: (**A**) A site; (**B**) B site; (**C**) C site; (**D**) D site. (Base A is red, base T is green, and GOQDs are in blue).

Based on the simulation results, the possible adsorption sites of various GOQDs on the DNA fragments are shown in Figure 3. The adsorption sites on the DNA fragments could be divided into four sites (A, B, C, and D). Site A is the head of the DNA fragment. At site A, the benzene ring of the GOQDs is parallel or vertical to the pyrimidine ring of the starting base in poly(A−T)_20_. In vertical absorption, GOQDs can insert and break hydrogen bonds between A and T. The absorption of GOQDs on the first minor groove of the DNA fragment was defined as the B site absorption. The GOQDs molecules that entered the groove of the DNA molecule were captured. In these types of absorption, GOQDs can significantly affect the length of the DNA fragment when aggregated into a cluster. The C site is another minor groove of the DNA fragment; thus, the mode of interaction between the GOQDs and DNA is similar to that of the B site. The D site is the terminal end of the DNA fragment. At this site, the interaction between the GOQDs and the DNA fragment is similar to that at site A.

**Figure 4:**
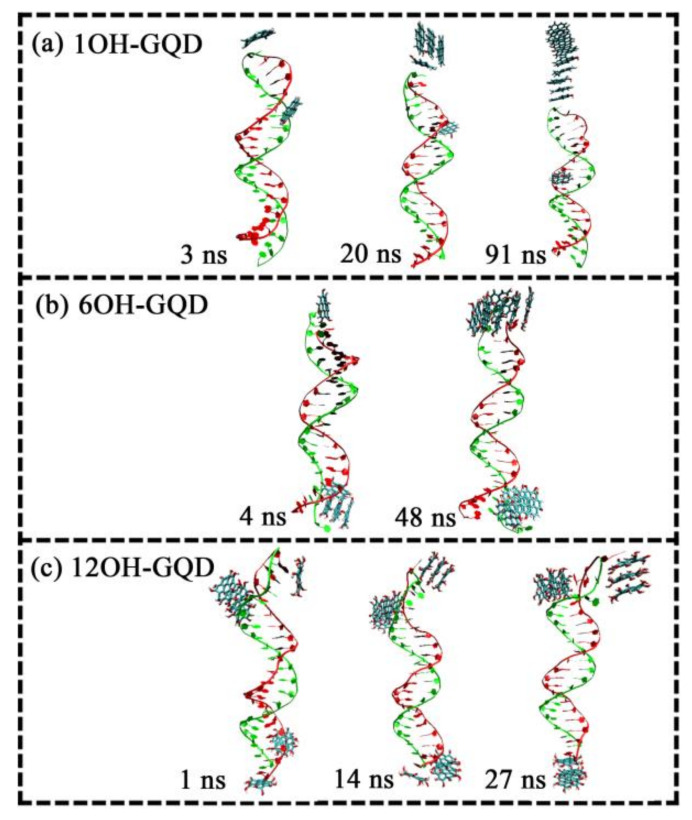
Absorption of GOQDs with different degrees of oxidation on DNA fragment at a concentration of 10 at a simulation time of 100 ns: (**a**) 1OH-GQD; (**b**) 6OH-GQD; (**c**) 12OH-GQD. (Base A is red, base T is green, and GOQDs are in blue).

A system with moderate concentration with 10 GOQDs was used as an example to analyze the influence of the degree of oxidation on the adsorption site on DNA. As can be observed from the snapshots of the trajectory, 1OH-GQDs have two adsorption sites on DNA, A and B, as shown in Figure 4a. Five 1OH-GQDs were adsorbed on the DNA fragment after a simulation for 20 ns. After 70 ns, all the GOQDs were adsorbed onto the DNA fragment. One 1OH-GQD was adsorbed on the B site, and nine 1OH-GQDs were adsorbed on the A site. The plane of the GOQDs was parallel to that of the A−T bases at site A. At site B, the 1OH-GQD could be inserted into the interior of poly(A−T)_20_ and break the hydrogen bond between A and T bases, leading to a mismatch of A−T of the DNA molecule. Only one 1OH-GQD entered the interior of the DNA molecule, indicating that the ratio that interacted with the minor groove was very low. The 6OH-GQDs had two adsorption sites on DNA, including the A and C sites, as shown in Figure 4b. Four 6OH-GQDs adsorbed on DNA fragments after simulation for 4 ns. After 44 ns, all the 6OH-GQDs were adsorbed onto the DNA fragment. Six 6OH-GQDs were adsorbed at the A site, and four 6OH-GQDs were adsorbed at the C site. In addition to the difference in the adsorbed number of GOQDs, compared with the parallel adsorption of 1OH-GQDs, the adsorption of the 6OH-GQDs showed a tendency to insert between the base pairs at the A site. Additionally, we established that base pair A1-T20 opened, and the two bases flipped out at the C site; however, the T20 base paired with the 6OH-GQD and base A1 flipped out the fragment, which was similar to the interaction of doxorubicin with the DNA fragment [49,50]. As shown in Figure 4c, the adsorption sites of the 12OH-GQDs on the DNA fragment increased to three. Seven 12OH-GQDs initially adsorbed onto the DNA fragment. After 14 ns, all the GOQDs were adsorbed on the DNA fragment, and the GOQDs adjusted their position from the C site to the D site; thus, the adsorption sites on DNA changed from four to three. Therefore, three 12OH-GQDs were adsorbed at the A site, four 12OH-GQDs were adsorbed at the B site, and three 12OH-GQDs were adsorbed at the D site.

**Figure 5:**
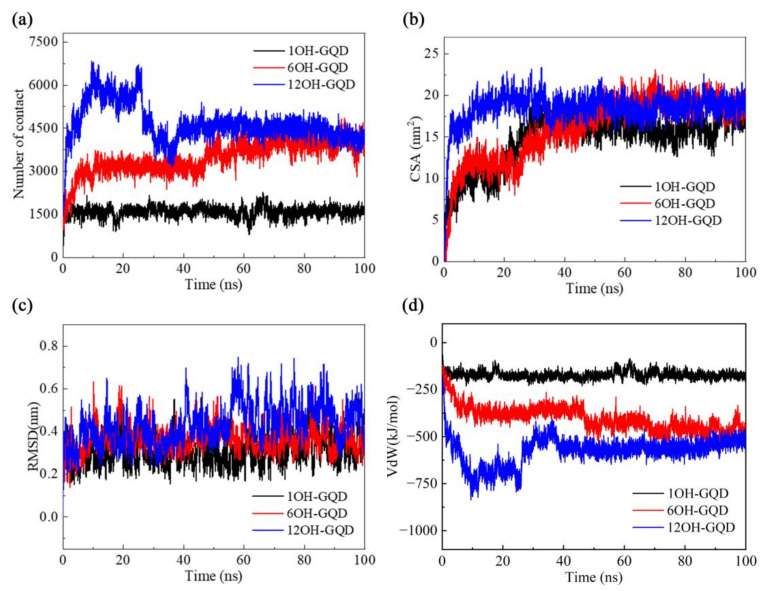
(**a**) Number of contacts between DNA and various GOQDs; (**b**) CSA between DNA and GOQDs; (**c**) RMSD of DNA as a function of time; (**d**) VdW energy between DNA and various GOQDs.

In order to quantitatively characterize the adsorption process observed in the trajectories, we calculated the number of atom contacts and the contacting surface area (CSA) between the DNA and various GOQDs. Contact is defined if the distance between an atom of DNA and that of GOQDs is less than 0.6 nm, and CSA is defined according to the following formula:

CSA = (SASA_DNA_ + SASA_GOQDs_ − SASA_DNA-GOQDs_)/2,

where SASA is the solvent-accessible surface area. As shown in Figure 5b, in the 1OH-GQDs system, CSA increased from 0 to 5 nm^2^ in 3 ns, and it continued to increase to 18 nm^2^ in 32 ns. Finally, the CSA had a slight increase in 89 ns, and the number of contacts was stable during the entire adsorption process. This is because the 1OH-GQDs had only two adsorption sites in the DNA fragment, as shown in Figure 4a. Thus, the number of contacts between 1OH-GQDs and DNA was steady from the beginning, and the CSA remained unchanged because of the self-aggregation of 1OH-GQDs in water. In the 6OH-GQDs system, the number of contacts increased from 0 to 3000 in 5 ns and remained stable until 48 ns; it then increased to 3800 until the end, which was consistent with the adsorption process, as shown in Figure 4b. CSA increased from 0 to 12 nm^2^ over 5 ns and remained steady until 25 ns. Thereafter, accompanied by the aggregation of the 6OH-GQDs, CSA increased to 18 nm^2^ until 50 ns and was maintained at 18 nm^2^. In the 12OH-GQDs system, this corresponded to a rapid adsorption process; thus, CSA immediately increased from 0 to 17 nm^2^ in 4 ns. Thereafter, it gradually increased to 20 nm^2^ in ca. 12 ns and continued until the end. The number of contacts clearly reflects the position adjustment of the 12OH-GQDs during the adsorption process on DNA. The number of contacts increased to 6000 in 10 ns. However, from 25 ns to 35 ns, the number of contacts decreased to 4500, and CSA decreased slightly in 35 ns, corresponding to the adsorption sites of 12OH-GQDs on DNA changed from four to three, as shown in Figure 4c. Figure 5a shows that the root mean square deviation (RMSD) of DNA in the three systems experienced various fluctuations and converged to 0.32, 0.38, and 0.48 nm (average over the last 50 ns in all independent trajectories). The RMSD of the DNA in the system of 12OH-GQDs is the largest. In order to illustrate the conformational changes in DNA in detail, we calculated the Van der Waals interaction energy (VdW) between DNA and various GOQDs. As shown in Figure 5d, there was a rapid increase in the DNA–GOQDs interaction energy from 0 ns to 10 ns in the 12OH-GQDs system. In the 6OH-GQDs system, the interaction energy between DNA and 6OH-GQDs slowly increased from 0 to 50 ns, followed by a small increase that remained stable. In the 1OH-GQDs system, within the first few nanoseconds, the VdW interaction energy remained stable within the first few nanoseconds. The change in VdW energy between the DNA and GOQDs was similar to the number of contacts. As shown in Figure S1, the VdW energy has an approximately linear relationship with the number of contacts. The results indicated that the interaction between the GOQDs and DNA was dominated by the VdW energy.

**Figure 6:**
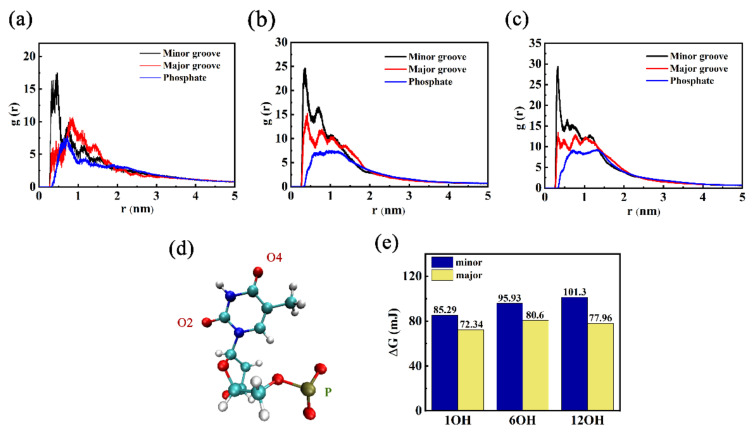
Average radial distribution functions (RDFs) for GOQDs-O present in the simulations and three RNA atoms, (P (blue), T-O2 (black), and T-O4 (red)): (**a**) 1OH-GQD; (**b**) 6OH-GQD; (**c**) 12OH-GQD; (**d**) the structure of thymine; (**e**) Gibbs energy of activation for contact breaking between DNA atoms and GOQD-O.

In order to understand the preferred binding site of DNA in the groove, the g(r) of the OH group (GOQDs-O, hydrogen bond donor site) of various GOQDs with phosphate (P), minor groove (T-O2), and major groove (T-O4) of DNA were calculated, as shown in Figure 6b–d. The g(r) of the GOQDs with DNA clearly indicated that the GOQDs preferred to accommodate minor grooves compared to the major groove and P backbone. Conversely, the g(r) of HCQ-O with DNA showed that as the degree of oxidation increased, the possibility of interaction of the OH group with the major groove and P backbone of DNA was significantly less than that of the minor groove. The Gibbs activation energies were also compared. This result is consistent with that of g(r). Herein, an analysis method developed to study the kinetics of hydrogen bond breaking and forming and the thermodynamics of hydrogen bond breaking in different environments was used to study the binding energetics [51,52,53]. This method yielded the Gibbs energy of dG for contact breaking, and it was previously applied to RNA-ion contact in a study of viral RNA [54]. dG was defined according to the following formula:

$$\mathrm{dG}=K_{B}T\ln\frac{g{\left(r\right)}_{\max}}{g{\left(r\right)}_{\infty }}$$

As mentioned above, with a high concentration of GOQDs, a higher oxidation degree indicated more adsorption sites on DNA, and the time that all the GOQDs adsorbed on DNA was significantly reduced. However, as the degree of oxidation increased, the interaction of the OH group with the minor groove of DNA also increased. Thus, it had a significant effect on the DNA structure in the 12OH-GQDs system. This is consistent with our previous research. In a past study, we explored the effect of the size of GQDs on DNA damage and found that the influence of small-size GQDs on the DNA is bigger than that of large-size GQDs, and they can even destroy the double helix structure by insertion into the interior of the base pair of the DNA fragment. This part of the study was completed by analyzing the interaction of small-size GOQDs with different degrees of oxidation with DNA.

### 3.2. Effects of Different Oxidation Degrees of GOQDs on DNA Damage

**Figure 7:**
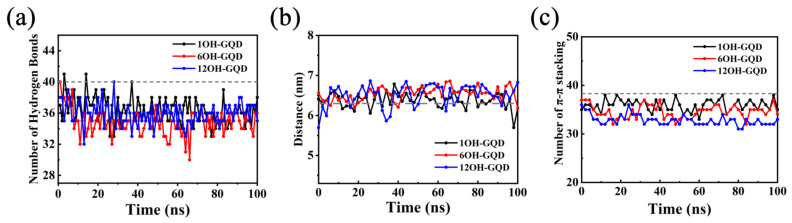
(**a**) Number of hydrogen bonds in DNA as a function of simulation time; (**b**) length of DNA fragments as a function of simulation time; (**c**) number of intra-DNA π-π stacking as a function of time.

In order to elucidate the stability of DNA during the absorption of GOQDs, the number of hydrogen bonds in the DNA fragment was calculated during absorption. Before interacting with the GOQDs, 40 hydrogen bonds were formed within the DNA between the bases of the DNA double strand. After absorption by GOQDs, the number of hydrogen bonds in the DNA decreased. This implied that the base pairs between the DNA strands were broken and the DNA was damaged. As shown in Figure 7a, the number of hydrogen bonds in DNA in the three systems decreased, but the change from 1OH-GQD to 12OH-GQD was small. In the 6OH-GQD system, the number of hydrogen bonds was less than that in the other systems. The length of the DNA was measured to verify further the damage caused by GOQDs to DNA. DNA length was defined as the distance between the centroid of the two bases at the top and those at the bottom. Changes in the DNA length indicate the stability of the double helix structure on the DNA chain. As shown in Figure 7b, the length of DNA in the 6OH-GQD and 12OH-GQD systems increased from 6.2 to 6.6 nm (average over the last 30 ns in all independent trajectories). In the 1OH-GQD system, the length of the DNA only changed from 6.2 to 6.3 nm. This indicated that the adsorption of 6OH-GQD in the minor groove of DNA caused the unwinding of the DNA double helix structure. This could lead to the elongation of DNA and the destruction of hydrogen bonds between bases at the adsorption position. In addition, we calculated the number of intra-DNA π-π stacking. If the distance between the centroid of the two aromatic rings of the base pairs was less than 4 Å and the angle of the planes of the two rings was less than 36°, π-π interactions were considered in the two aromatic rings [44]. As shown in Figure 7c, the number of intra-DNA π-π stacking remained steady. The number of π-π stacking decreased to 32 in the 12OH-GQD system, followed by 34 in the 6OH-GQD system, and 36 in the 1OH-GQD system (average over the last 30 ns). It also reflects the length of DNA fluctuation in the 12OH-GQD system because the number of π-π stacking was reduced between the base pairs.

The above analysis indicated that the number of π-π stacking is inversely proportional to the oxidation degree of the GOQDs. The oxidation degree of GOQDs showed a slight difference in the number of hydrogen bonds in DNA, and the destruction of hydrogen bonds in DNA depended on the interaction mechanism that GOQDs acted with DNA.

### 3.3. Effects of the Number of GOQDs on DNA Damage

**Figure 8:**
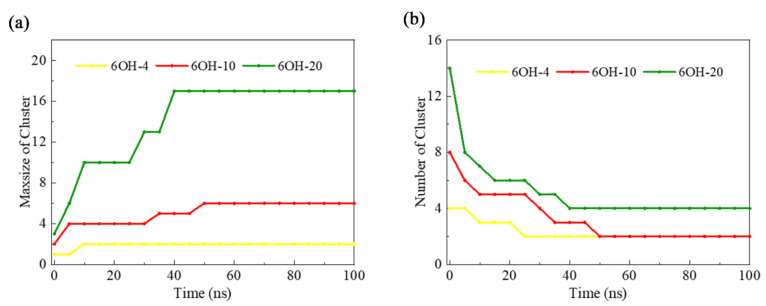
(**a**) Number of clusters and (**b**) the maximum cluster as a function of the simulation time.

In order to further uncover the effect of GOQD concentration on DNA damage, simulations with different numbers of 6OH-GOQDs were performed. As shown in Figure S3, the number of hydrogen bonds in the DNA in the AT-6OH-10 system was less than those in the AT-6OH-4 and AT-6OH-20 systems. The AT-6OH-10 system had the longest DNA. The change in the number of π-π stacking was greatest in the AT-6OH-10 system. From the trajectory analysis, we found four adsorption sites on the DNA in the AT-6OH-20 system, as shown in Figure S2. Thus, the number of π-π stacking was the most affected. The g(r) in the AT-6OH-20 system, as shown in Figure S2d, shows that with an increase in the GOQDs concentration, the g(r) of the minor groove decreased compared with that of the AT-6OH-10 system. GOQDs aggregated into a cluster and were adsorbed on the D site on the terminal of DNA. The big cluster was hard to enter into the minor groove of DNA, as shown in Figure S2c. This was verified by counting the number of GOQD clusters and the number of GOQDs in the largest cluster, as shown in Figure 8. It can be observed that in the 100 ns simulation, the number of clusters in the AT-6OH-20 and AT-6OH-10 systems decreased to 4 and 2, respectively, whereas the number of GOQDs in the largest maximum cluster in the AT-6OH-20 system reached 17, and that in the AT-6OH-10 system was only 5.

From the above discussion, we established that the toxicity of the 6OH-GQDs did not increase with concentration. Although it had oxide groups at the edge, the GOQDs preferred to aggregate together in a large cluster and adsorb on the DNA terminal when the number of 6OH-GQDs was 20. When the number of 6OH-GQDs was 10, the GOQDs aggregated to a small cluster and could be inserted in the minor groove on DNA, showing more toxicity.

### 3.4. Effect of Different Functional Groups of GOQDs on DNA Damage

In order to elucidate the effect of the different functional groups of the GOQDs on the adsorption of DNA fragments, simulations with different functional groups -1COOH were performed, as listed in Table 1. As shown in Figure S4, there were three adsorption sites on DNA, including sites A, B, and D. There was one 1COOH-GQD at site A, two 1COOH-GQDs at site B, and seven 1COOH-GQDs at site D. We characterized the adsorption process between DNA and 1COOH-GQDs. As shown in Figure S5, the number of contacts increased from 1200 to 2500 in 30 ns and then decreased slightly at 50 ns. The CSA reflected the two moments that increased from 3.8 to 7.5 nm at 30 ns and decreased from 10.1 to 7.5 nm at 50 ns. However, the RMSD data showed that the DNA structure was affected the most at 10 and 50 ns. The snapshot of the 1COOH-GQDs with DNA in Figure S4 shows that the 1COOH-GQDs contacted the DNA in 10 ns, and the RMSD only increased slightly. This indicates that the 1COOH-GQDs were adsorbed with the P skeleton and deformed the DNA structure. The 1COOH-GQDs entered the minor groove on the DNA at 20 ns, the same as at 50 ns. Another finding was that the position of the 1COOH-GQDs in the minor groove at 20 ns was different from the position at 67 ns. This means that the 1COOH-GQDs could move in the minor groove of DNA, suggesting that the binding between them was weak.

As shown in Figure S6, the number of hydrogen bonds and π-π stacking in DNA were more in the 1COOH-GQDs system than in the 1OH-GQDs system. The length of the DNA was more than the 1OH-GQDs system. From the RDF data, we can observe that the g(r) of O1 and O2 in the 1COOH-GQD system in the minor groove on DNA was larger than that in the 1OH-GQD system. This suggests that the binding between them is stronger than the 1OH-GQDs system.

The above analysis indicated that although the binding between the 1COOH-GQDs in the minor groove on DNA was stronger than the 1OH-GQDs system, the effect on DNA was weaker. This indicates that the 1COOH-GQDs are less toxic. However, the current research has some shortcomings. For example, there is a lack of relevant experimental verification of whether these functional groups have the same effect on other materials when they interact with biological molecules. In the following work, on the one hand, we will combine more experiments to summarize the interaction mechanism so as to better interpret the molecular mechanism of the interaction between nanomaterials and biomolecules. On the other hand, explore the possible practical application of GOQDs.

## 4. Conclusions

By conducting simulations on various GOQDs with different concentrations and oxidation degrees, we uncovered four absorption sites of GOQDs on the DNA fragment, including the head, tail, and two grooves of the DNA fragment. The adsorption of GOQDs in the minor groove has a great effect on the structure of DNA. In the case where the adsorbed GOQDs are small, the GOQD can insert into the interior of the base pair and break the hydrogen bond between the complementary base pair and the π-π stacking between the adjacent base pair. However, when there are more GOQDs in the minor groove of DNA, the base pairs at the adsorption sites will be open directly, which can cause serious damage to the double helix structure of DNA. The linear correlation between the number of contact atoms and Van der Waals forces shows that the interaction between GOQDs and DNA is dominated by Van der Waals forces. In addition, we also investigated the effect of GOQDs carrying different functional groups on DNA. The results of this study may be conducive to the preparation, selection, and application of GOQDs with low toxicity in the biomedical field.

## Figures and Tables

**Table 1:** Details of all performed systems in this study.

System	Type of GOQD	Number of GOQDs	Number of Atoms	Simulation Time (ns)
AT-1OH-10	1OH-GQD	10:1	131,543	100
AT-6OH-10	6OH-GQD	10:1	104,140	100
AT-12OH-10	12OH-GQD	10:1	104,281	100
AT-6OH-4	6OH-GQD	4:1	89,383	100
AT-6OH-20	6OH-GQD	20:1	94,915	100
AT-1COOH-10	1COOH-GQD	10:1	104,392	100

## Supplementary Materials

The following supporting information can be downloaded at: https://www.mdpi.com/article/10.3390/ma15238506/s1, Figure S1: The relationship between the number of contacts and the VdW energy. (a) GQD-1OH, (b) GQD-6OH, (c) GQD-12OH; Figure S2: Adsorption sites of different number of GOQDs on DNA at the simulation time of 100 ns (a)The number of 6OH-GQDs is 4. (b) The number of 6OH-GQDs is 10. (c) The number of 6OH-GQDs is 20. (d) RDF for GOQDs-O present in the simulations and three DNA atoms (P(blue), T-O2(black), T-O4(red)) in AT-6OH-20 system; Figure S3: (a) Number of hydrogen bonds in DNA as a function of simulation time. (b) Number of intra-DNA π-π stacking as a function of time. (c) Length of DNA fragments as a function of simulation time; Figure S4: (a) Absorption of 1COOH-GQDs on DNA fragment at a concentration of 10 at the simulation time of 100 ns. (b) RDF for GOQDs-O1 and GOQDs-O2 present in the simulations and three DNA atoms (P(blue), T-O2(black), T-O4(red)); Figure S5: (a) The number of contacts between DNA and 1COOH-GQDs. (b) CSA (contact surface areas) between DNA and 1COOH-GQDs. (c) Root mean square deviation (RMSD) of DNA as a function of time. (d) The VdW energy between DNA and 1COOH-GQDs. (Black line is 1OH-GQDs, pink line is 1COOH-GQDs); Figure S6: (a) Number of hydrogen bonds in DNA as a function of simulation time. (b) Number of intra-DNA π-π stacking as a function of time. (c) Length of DNA fragments as a function of simulation time.
