# Supplementary material for: Molecular Dynamics Simulation of the Interaction between Graphene Oxide Quantum Dots and DNA Fragment
Source: Materials (Basel). 2022 Nov 29;15(23):8506. doi: 10.3390/ma15238506 (PMC9737461; doi:10.3390/ma15238506)
Supplement: Supplementary file 1 [file materials-15-08506-s001.zip › materials-2003007-supplementary.pdf]

Molecular dynamics simulation of the interaction between Graphene oxide Quantum dots and DNA fragment SI

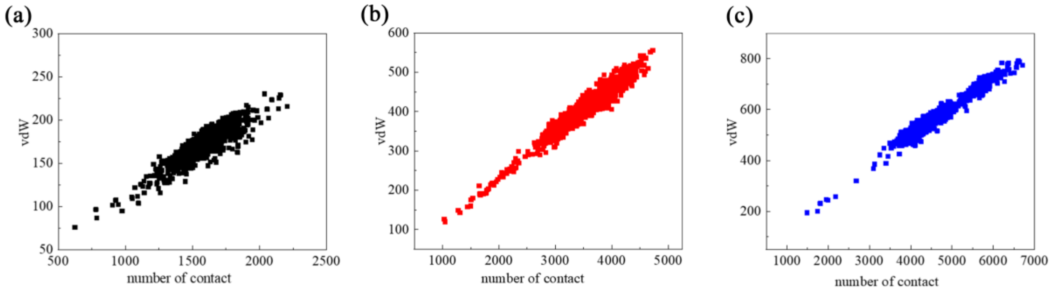

Figure S1: The relationship between the number of contacts and the VdW energy. (a) GQD-1OH, (b) GQD-6OH, (c) GQD-12OH.

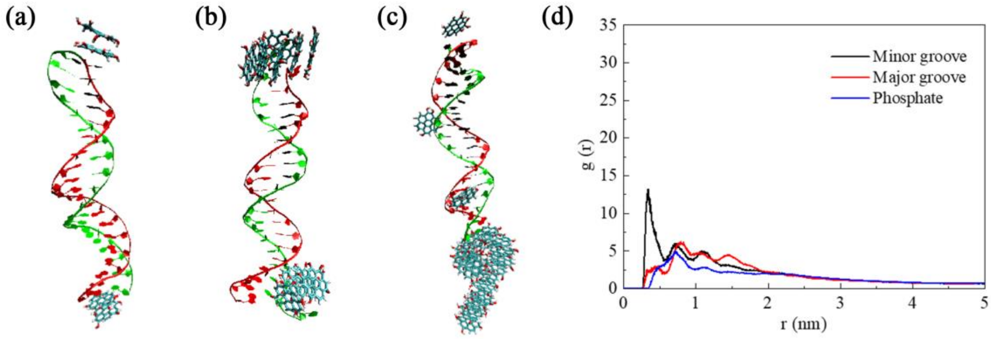

Figure S2: Adsorption sites of different number of GOQDs on DNA at the simulation time of 100 ns (a)The number of 6OH-GQDs is 4. (b) The number of 6OH-GQDs is 10. (c) The number of 6OH-GQDs is 20. (d) RDF for GOQDs-O present in the simulations and three DNA atoms (P(blue), T-O2(black), T-O4(red)) in AT-6OH-20 system.

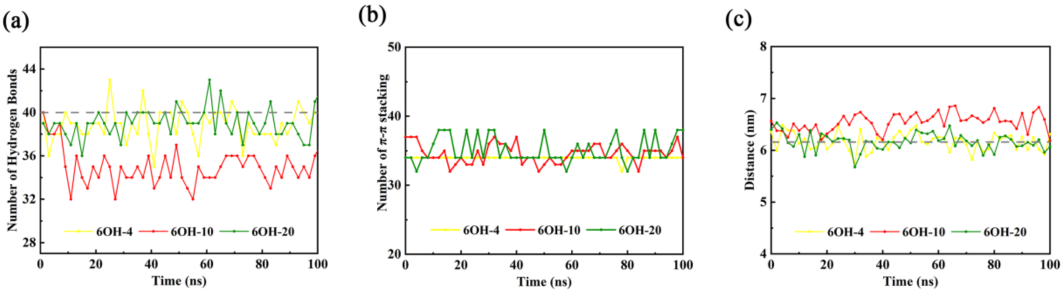

Figure S3: (a) Number of hydrogen bonds in DNA as a function of simulation time. (b) Number of intra-DNA π-π stacking as a function of time. (c) Length of DNA fragments as a function of simulation time.

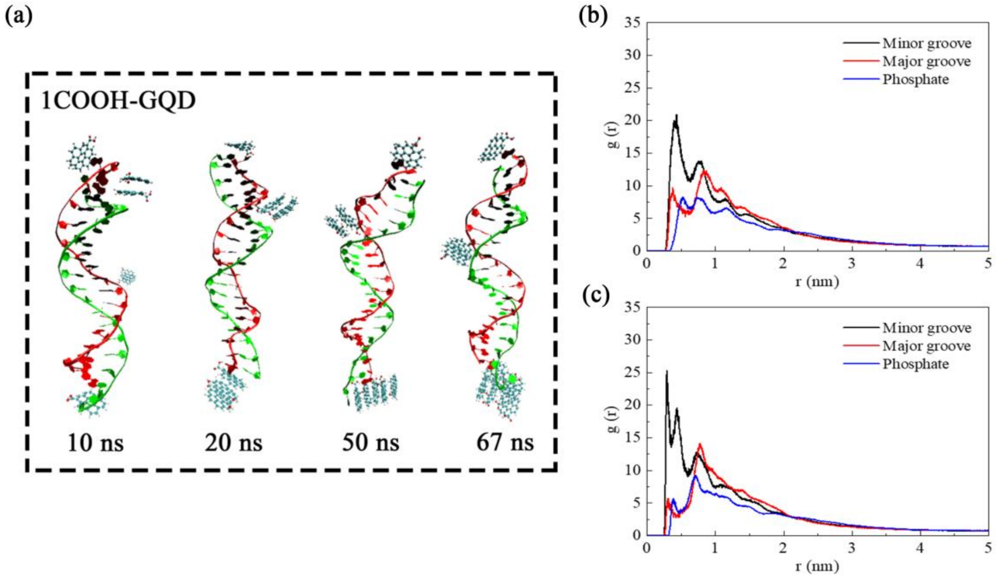

Figure S4: (a) Absorption of 1COOH-GQDs on DNA fragment at a concentration of 10 at the simulation time of 100 ns. (b) RDF for GOQDs-O1 and GOQDs-O2 present in the simulations and three DNA atoms (P(blue), T-O2(black), T-O4(red)).

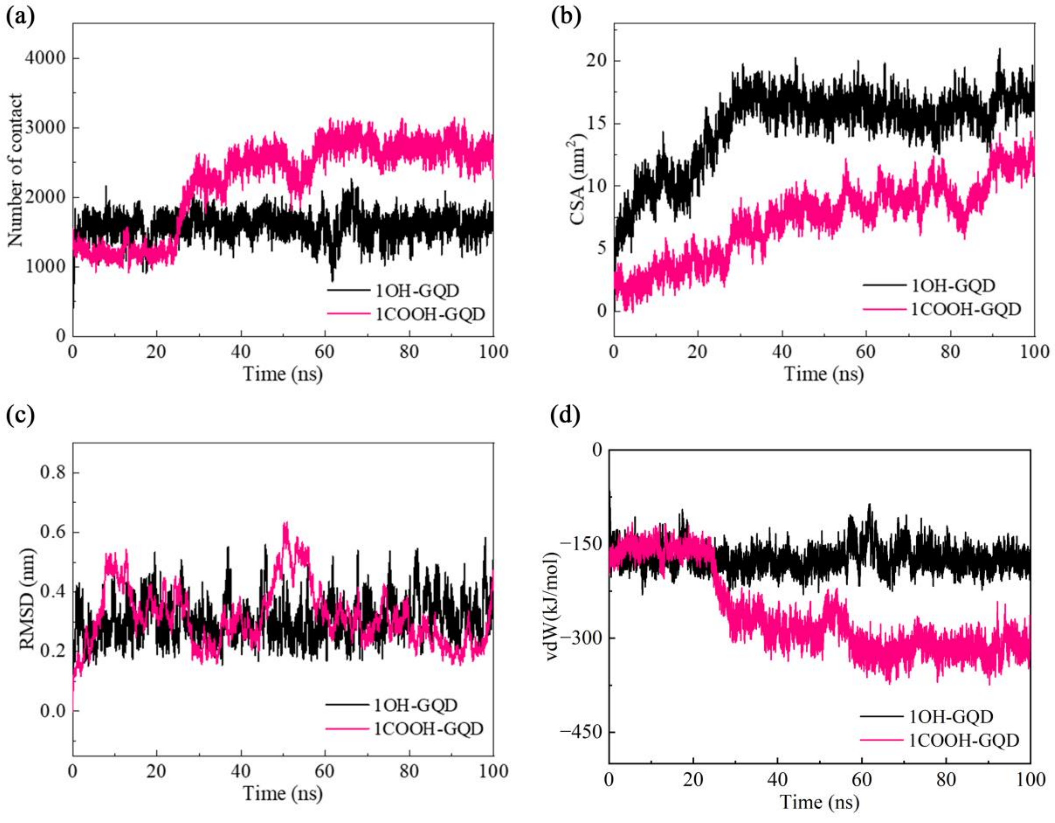

Figure S5: (a) The number of contacts between DNA and 1COOH-GQDs. (b) CSA (contact surface areas) between DNA and 1COOH-GQDs. (c) Root mean square deviation (RMSD) of DNA as a function of time. (d) The VdW energy between DNA and 1COOH-GQDs. (Black line is 1OH-GQDs, pink line is 1COOH-GQDs)

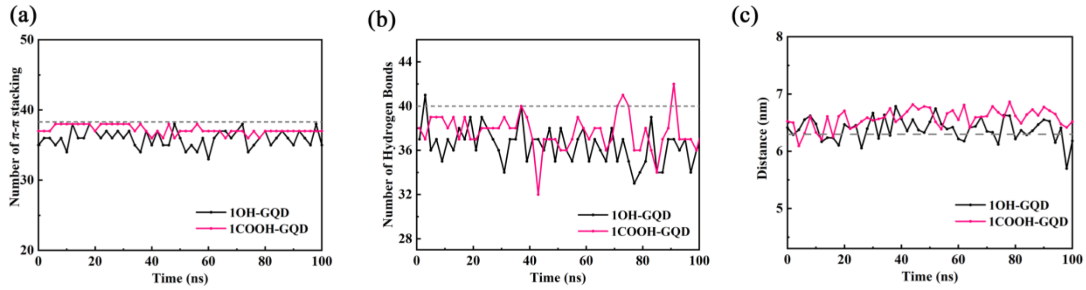

Figure S6: (a) Number of hydrogen bonds in DNA as a function of simulation time. (b) Number of intra-DNA π-π stacking as a function of time. (c) Length of DNA fragments as a function of simulation time.
